# Association between Parenthood and Health Behaviour in Later Life—Results from the Population-Based CARLA Study

**DOI:** 10.3390/ijerph19010082

**Published:** 2021-12-22

**Authors:** Lisa Becker, Sarah Negash, Nadja Kartschmit, Alexander Kluttig, Rafael Mikolajczyk

**Affiliations:** Institute of Medical Epidemiology, Biometry and Informatics, Martin-Luther-University Halle-Wittenberg, Magdeburger Str. 8, 06112 Halle, Germany; lisa.becker@uk-halle.de (L.B.); sarah.negash@uk-halle.de (S.N.); nadja.kartschmit@uk-halle.de (N.K.); alexander.kluttig@uk-halle.de (A.K.)

**Keywords:** parenthood, childlessness, health behaviour, physical activity, diet, smoking, alcohol, ageing

## Abstract

Previous research has focused on comparing health behaviour between parents and non-parents at younger ages, while little is known about the impact of being a parent on health behaviours in later life. We studied whether parenthood is associated with later physical activity (PA), dietary pattern, smoking status and alcohol consumption in German adults of middle and old age. We used data from the baseline examination of the population-based CARLA-study in Halle (Saale), comprising 1779 adults aged 45–83. Linear and logistic regression analyses assessed the relationship between parenthood and health behaviours while controlling for age, partner status, education, income, occupational position, socioeconomic status in childhood, and number of chronic diseases. Of the participants, 89.1% had biological children. Being a father was associated with higher PA in sports (sport index ß = 0.29, 95% confidence interval [0.14; 0.44]), but not with PA in leisure time (excluding sports), dietary pattern, consumption of alcohol and smoking status. No associations were found between being a mother with all outcome variables. Provided that PA of fathers is typically reduced when the children are young, the development towards higher PA at later age needs to be studied in more detail.

## 1. Introduction

Engaging in healthy behaviours, such as being physical activity, eating a wholesome diet and avoiding tobacco and alcohol consumption, plays a pivotal role in health. Evidence suggests that these behaviours, especially when combined, lead not only to improved longevity [1,2] but also to a longer life in good health [3,4].

From a life-course perspective, parenthood is a major life event. Regarding health behaviour, becoming a parent can be a critical turning point with the ability to shape health trajectories later in life [5,6,7]. Previous research has focused often on health behaviour among parents of young children. When compared to their childless counterparts, parents spent less time in moderate to vigorous physical activity [8,9,10,11,12,13,14,15]. In terms of nutrition, some studies reported a slightly higher intake of energy and fat among parents [16,17], whereas other studies found no difference in dietary pattern between parents and childless individuals [11,18]. Research regarding the association of parenthood and substance abuse reported a lower prevalence of smoking [10,19,20,21] and a decreased consumption of alcohol [22,23,24,25] among parents of young children. Kendig et al. investigated parents’ health-related lifestyles in an older population and suggested more favourable health behaviours in older parents when compared to childless adults [7]. However, following the fact that unstandardised outcome variables were used and the analyses were only adjusted for age and education [7], more research in elderly populations is strongly required, especially since data from the “German Health Update” (GEDA) showed that the association between living with children and health behaviours varied with the parents’ age. The authors found that parenthood (defined as living with children) was associated with adverse health behaviours in younger adults, whereas parents between the ages of 45 and 54 behaved more healthily than non-parents [14]. Another study that compared the health of parents with children of different ages showed that parents whose youngest child was 30 years and older had higher levels of life satisfaction than non-parents and better physical and mental health outcomes than parents of younger children [26]. This may be caused by a decrease in financial, emotional and time burden when children reach adulthood, resulting in a predominance of positive effects of parenthood, such as improved social support and a sense of meaning in life [26,27]. The theory that the rewards of parenthood outweigh its demands in later life [27] is in accordance with studies in elderly populations (aged 45 and older), reporting better mental health [28,29], lower prevalence of cardiovascular [30,31,32] and metabolic diseases [33] and lower mortality rates [32,34,35] among those who have had biological children in comparison to those who remained childless. The explanation for the differences in health was considered to result from lifestyle differences [31,36,37,38,39]. Still, the knowledge of health behaviour differences in later life of parents versus non-parents is limited to the two cited studies [7,14].

It is important to gain knowledge on whether the life transition to parenthood, that affects a major part of the population across their life courses, impacts key modifiable health behaviours for the prevention of non-communicable diseases (NCDs), especially in ageing societies [40,41,42]. Therefore, this study aims to determine whether physical activity levels, dietary pattern, tobacco and alcohol consumption in later life differ between parents and childless individuals in a German population of middle and old ages.

## 2. Materials and Methods

### 2.1. Study Population

For the analysis, data from the baseline examination of the population-based cohort study *CARdiovascular Disease, Living and Ageing in Halle* (CARLA) in Halle (Saale), a city in the eastern part of Germany, were used. The baseline assessment took place between 2002 and 2006. A total of 1779 adults (812 women, 967 men) between the ages of 45 and 83 took part at baseline with a response proportion of 64% (60% women, 69% men). To assess non-response bias, a telephone interview was carried out among 373 of the non-responders. Data were collected using a physical examination and a standardised, computer-assisted interview by a trained study nurse as well as self-administrated questionnaires. Thus, information on socio-demographic and socio-economic status, biomedical, behavioural and psychological factors and medical history were assessed. More detailed information about the study is published elsewhere [43,44].

In all cases, participants’ written consent was obtained and the study protocol was approved by the Ethics Committee of the Martin Luther University Halle-Wittenberg (Halle (Saale), Germany).

### 2.2. Parental Status

For the assessment of the parental status, participants were asked to name the number of their biological children. Consequently, all women and men were categorised into non-parents (0 children) and parents (≥1 children).

### 2.3. Health Behaviour

The present analysis includes self-reported measures of physical activity, dietary pattern index, alcohol consumption and smoking status as outcome variables.

Physical activity during the last 12 months was assessed using the Baecke questionnaire [45,46]. It provides information about physical activity levels in two main categories: sports-related physical activity (sport index) and physical activity in leisure-time other than sports (leisure-time index). For each of the two modes of physical activity, a particular index indicating an activity level from 1 (low) to 5 (high) was calculated. It is based on five questions regarding the intensity, frequency and duration of the self-reported practised activities. We further included the binary variable of being active in sports (yes vs. no) in our analysis.

To assess the dietary pattern during the last 12 months, a validated food frequency questionnaire was applied, which allows to calculate the dietary pattern index [47]. The dietary pattern index indicates a favourable dietary pattern, characterised by frequent consumption of (whole-grain) carbohydrates, vegetables and fruits. Frequencies of consumption of selected food groups were used to calculate the index score, ranging from 0 (worst dietary pattern) to 30 (best dietary pattern) [48].

In order to quantify the alcohol consumption, participants were asked to count the number of glasses of beer (0.5 L), wine or champagne (0.2 L) and spirits (2 cL/glass) they consumed per week on average. From that, we calculated the grams of alcohol each participant consumed per day, based on a concentration of alcohol of 4.8 volume-% for beer, 11.0 volume-% for wine and champagne, and 33.0 volume-% for spirits [49]. In our analysis, we included a binary variable, distinguishing between no consumption (0 g) and any consumption of alcohol (>0 g) per day.

With respect to smoking status, participants were classified into “current smoker”, “former smoker” or “never smoker”. Smoking was defined as consumption of tobacco products at least once a week for a minimum duration of 12 months. Participants were classified as former smokers if they had quit smoking for at least 12 months after consuming the aforesaid amount of tobacco products.

### 2.4. Confounders

We included the following variables as confounders for the association between being a parent and health behaviour: self-reported sex (male and female), age (in years), living with a partner (yes vs. no), current socioeconomic status as well as socioeconomic status in childhood, and the number of chronic diseases. We selected the confounders based on a thorough literature review (e.g., [50,51,52,53]).

To determine socioeconomic status, we included education, occupation, income, and socioeconomic status during the participant’s childhood as separate variables in the analyses. Years of education were classified according to the International Standard Classification of Education (ISCED, 1997), comprising both school and vocational education. Current and last occupational position, respectively, were grouped into three categories, including unskilled-simple occupation (blue-collar worker, farmer), qualified occupation (foreman, qualified employee, self-employed with <10 employees) and high qualified occupation (highly qualified employee, supervisor, self-employed with ≥10 employees, academic professions). In order to measure income, the monthly equivalent household net income per person was calculated and classified into three categories: (i) less than EUR 750, (ii) EUR 750 to less than EUR 1500, and (iii) EUR 1500 and more. In addition, the participant’s socioeconomic status in childhood was calculated from the education and occupational position of each parent, given a double weighting of the father’s values. In cases where information about only one parent was obtained, the variable was computed from this based on this parent only. Lastly, the number of chronic conditions was used as an ordinal variable ranging from “0” to “3 and more” self-reported physician-diagnosed chronic diseases.

### 2.5. Statistical Analysis

The frequencies or means with corresponding 95% confidence interval (CI) of the health behaviour variables were estimated for parents and non-parents. In order to investigate the associations between parenthood and sport index, leisure-time index and dietary pattern index, linear regression models were applied. Logistic regression models were used for the relationship between being a parent and the outcome variables ‘being active in sports’ (yes vs. no) and ‘consumption of alcohol’ (0 g vs. any consumption). The association of being a parent and smoking status was examined by performing multinomial logistic regression models. Firstly, all regression analyses were performed unadjusted. Secondly, we adjusted for age, partner status, education, occupational position, income, socioeconomic status in childhood and the number of chronic conditions. We stratified all analyses by sex. All analyses were carried out using IBM SPSS Statistics 25 (2017).

## 3. Results

Overall, 89.1% of the participants were parents (88.3% women, 89.8% men). Further details of the study participants are shown in Table 1.

**Figure 1:**
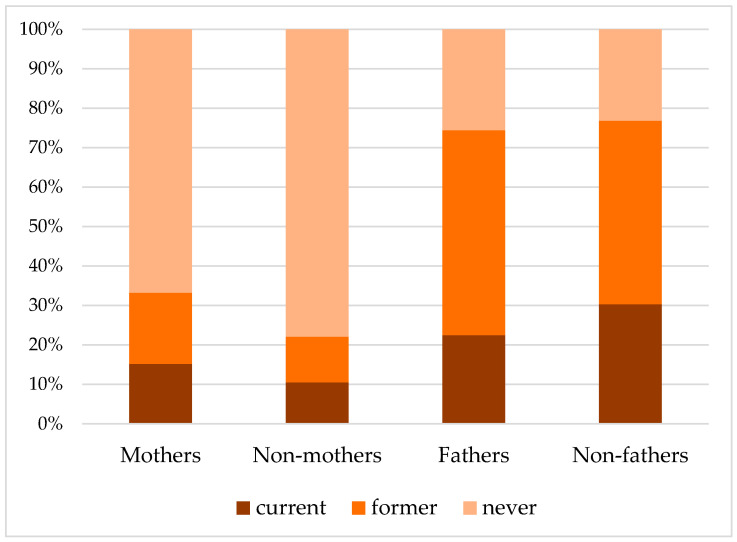
Current, former and never smoking, stratified by parental status and sex.

The proportion being active in sports was higher for mothers and fathers than for childless adults (Table 2). Over half of the women (55.0%) and 21.4% of men reported to consume 0 g alcohol per day. The proportion of drinking any alcohol was independent of parental status. When compared to childless women, mothers were more often current smokers (15.2% vs. 10.5%) and less often never smokers (66.6% vs. 77.9%) (Figure 1). Fathers, in turn, were less often current smokers (22.5% vs. 30.3%) and more often never smokers (25.6% vs. 23.2%) than childless men. The proportion of former smokers was higher among both mothers and fathers when compared to their childless counterparts.

The sport index of fathers was 0.29 points higher compared to non-fathers in adjusted regression analysis (CI: 0.14; 0.44) (Table 3 and Table 4). This means that fathers’ answers to one of five questions on sporting activities were at least one item higher than those of childless men. The difference between these both groups explained 1.4% of the total variance. No differences in sport index were found between mothers and non-mothers (ß: 0.10 (−0.06; 0.25)). The leisure-time index was not associated with parenthood for both women (ß: 0.06 (−0.07; 0.20)) and men (ß: 0.03 (−0.11; 0.16)). Fathers had higher odds to be active in sport than non-fathers (Odds Ratio (OR): 2.00 (1.14; 3.50)). In both sexes, there was no association between parenthood and dietary pattern (women ß: −0.05 (−0.76; 0.66), men ß: −0.04 (−0.71; 0.62)) as well as alcohol consumption (women OR: 0.84 (0.52; 1.36), men OR: 0.89 (0.52; 1.53)). Furthermore, we found no consistent association between being a parent vs. non-parent and smoking status for both, the odds of being a former vs. current smoker (women OR: 1.60 (0.61; 4.21), men OR: 1.35 (0.77; 2.38)) and of never vs. current smoking (women OR: 0.99 (0.46; 2.12), men OR: 1.45 (0.76; 2.78)).

## 4. Discussion

The present study investigated whether being a parent is associated with physical activity, dietary pattern and consumption of tobacco and alcohol in adults of middle and old age. Our results suggested that fatherhood is associated with later life sporting activities, but not with physical activity in leisure time other than sports. Being a father was not associated with dietary patterns, smoking and alcohol consumption. In women, no associations between parenthood and health-related lifestyles in later life were found.

### 4.1. Physical Activity in Sports

The results of this study indicate that fatherhood is associated with sport index and being active in sports in adults aged 45 and older. Higher levels of physical activity among elderly parents compared to non-parents were also reported by earlier studies [7,14], although this question has received scant attention in the previous literature. It is interesting to note that this result is contrary to research in younger parents, which has suggested decreased physical activity levels in parents when compared to their childless counterparts [8,9,10,11,12,13,15]. This contrast broadly supports the work of Rattay and von der Lippe linking the parents’ age with the direction of the association between parenthood and health behaviour [14]. Among parents of young children, this relationship may partly be explained by a lack of time due to family responsibilities that were reported to be a barrier to physical activity [54,55,56,57,58]. These time constraints may dwindle as children grow older and childcare responsibilities diminish. Furthermore, higher physical activity levels among elderly parents may be a result of improved social support. Previous research has established that older adults with children had more frequent social contacts [59,60] and received more social support [61,62] than their childless counterparts. A recent systematic review regarding social influence on physical activity levels reported that particularly social support from family members was positively associated with being physically active [63]. Nevertheless, other studies found no parental status differences in older adults’ social support [64,65]. Another explanatory theory is that adults with children may experience more social control, which can motivate parents to engage in healthier behaviours [6,7].

Another important finding was that associations between being a parent and sporting activities (sport index, being active in sports) were only observed in men. In line with our results, a cross-national analysis of health behaviours in elderly parents and childless individuals found a closer association between parenthood and physical activity in men than in women [7]. The gendered differences in parenthood-effects on physical activity levels may be explained by a different impact of social control on either sex. This view is supported by earlier studies that found stronger increases in men’s physical activity levels if they were married or had children when compared to women [7,58,66]. We found no association between parenthood and physical activity in sports among elderly women. Future studies are recommended to ascertain whether this finding occurred from the gendered differences in the impact of parenthood on later life sport participation or from our methodological approach. We used the same questionnaires for the physical activity assessment for women and men; however, the assessment of physical activity levels among women requires a more comprehensive approach than among men [67]. However, because our data were collected 20 years ago, gendered role perceptions may have changed in contemporary families [68] and further research is needed on gender differences in parents’ physical activity participation.

### 4.2. Physical Activity in Leisure-Time Other Than Sports

Unlike in the case of sporting activities, leisure-time activities other than sports did not seem to be influenced by parental status, neither in men nor in women. A possible explanation is that the types of physical activity included in the leisure–time index (i.e., walking, cycling) could be influenced to a higher extend by socioeconomic and environmental factors than sporting activities [69,70]. However, further research should be undertaken to investigate the relationship between parenthood and different modes of physical activity in later life.

### 4.3. Diet

This study did not find differences in dietary pattern between parents and non-parents in a population of middle and old age. A cross-sectional study that investigated the impact of parenthood on older adults’ fruit consumption in winter did not find marked differences between parents and non-parents [7]. Together with the absence of associations between being a parent and the validated dietary pattern index in our study, it could conceivably be hypothesised that older adults’ food choices may be less influenced by parental status. Since more detailed research on how parenthood along with its demands and rewards [27] influences eating habits throughout the parenting stages is lacking, further research on this issue is required.

### 4.4. Tobacco and Alcohol Consumption

Contrary to expectations, we did not find an association between being a parent and tobacco and alcohol consumption. Among adults of middle and old age, previous studies were not in line with our results by reporting a decreased consumption of tobacco and alcohol among parents over the age of 45 in comparison to their childless counterparts [7,14]. The findings from the “German Health Update” (GEDA), which compared health behaviours of parents and non-parents of different ages might not be in accordance with our results because of the definition of parents as adults living with children and the restriction to participants aged 18–54 [14]. It seems possible that the results of the study by Kendig et al. may differ from our results because the analyses were only adjusted for age and education and stratified by marital status [7]. In our analysis, we applied a more extensive adjustment set to minimise the confounding of socioeconomic disparities.

### 4.5. Limitations

Our results may be somewhat limited by the fact that all variables were self-reported and therefore susceptible to desirability and recall bias. Since the study design was cross-sectional, it is not possible to draw a causal interpretation of the results. It remains unclear whether having had children influences physical activity levels or whether personality traits leading to be generally more active have modified physical activity levels throughout the life course and therefore the opportunity to start a family. Nevertheless, there is some evidence that suggests that the causation hypothesis (health differences are a result of being a parent) is more likely than the selection of healthier adults into parenthood. A longitudinal study reported that the association of occupying multiple roles (employee, spouse, mother) with women’s better self-rated health in the age of 54 years was not explained by health status at baseline [71]. An additional uncontrolled factor is that some potential participants may have died prematurely due to causes related to parenthood. This might be especially the case in childless adults and in high-parity parents, because of higher mortality rates in these subgroups [34]. We did not include the number of children and the timing of births in our analyses. However, both high parity and early age at first birth are associated with disadvantageous later life health behaviours [36,37,38,72,73,74]. At the present time, alternative family constellations such as single parenthood, stepfamilies and LGBTQ parents gain in significance [27,75,76,77]. Albeit we were not able to include information on different types of families in our investigation, they may shape the impact of parenthood on health-related lifestyles and are important subjects for further research [77]. Due to the advanced age of our study population, however, it seems possible that the parents in this study rather lived in traditional family constellations. Moreover, this study was unable to investigate the impact of parenthood on additional behavioural outcomes, such as sedentary time, sleep behaviour or sexual risk behaviour, which could be affected by having had children. These are important issues for future research.

A further limitation of our study is that the data were gathered between 2002 and 2006 and thus reflect effects of parenthood in the preceding decades. They might not be transferable to the corresponding age groups in the present-day population. Lastly, the social context of our study cohort must be taken into account when considering its generalisability. Social norms and cultural values regarding parenthood are closely linked to its effect on health [78,79], which highlights the importance of considering the respective context. In particular, whether parents lived in East or West Germany has been found to affect how parenthood is associated with physical and mental health [80]. Our study, therefore, represents the former East German population and research in other parts of Germany as well as in other countries are required to contrast possible differences in health behaviours between elderly adults with and without children.

## 5. Conclusions

Our study showed that parenthood was associated with sporting activity in later life in men, but not in women. Being a parent was not associated with elderly adults’ leisure-time physical activity (excluding sports), dietary pattern, smoking status and alcohol consumption in both women and men. Our study demonstrated the urgent need to gain a deeper insight into the impacts of having children on behavioural decisions in later life. Our findings suggest that elderly childless men might especially be considered more for improving sport participation at the population level, however, further research is needed to explore gendered differences in the impact of having had children on physical activity in later life. Future research should also focus on health behaviours throughout the stages of parenthood by taking the ages of children into account and using a longitudinal study design to understand differences between parents’ and non-parents’ lifestyle trajectories over their life courses.

## Figures and Tables

**Table 1:** Characteristics of the study population (% or mean and SD), N = 1779.

Variable	Women	N	Missing (%)	Men	N	Missing (%)
	45.6%	812	0	54.4%	967	0
**Parents**	88.3%	717	0	89.8%	868	0
**Number of children**	2.0 (1.0)	717	11.7	2.1 (1.1)	868	10.2
**Age**	63.8 (9.9)		0	64.9 (10.2)		0
**Living with a partner**	61.1%	496	0	86.5%	836	0
**Education years**	13.93 (2.5)		0	15.21 (2.5)		0
**Occupational position**			0.7			0.3
Unskilled-simple	37.7%	304		38.2%	368	
Qualified	47.4%	382		25.9%	250	
High qualified	14.9%	120		35.9%	346	
**Net equivalent income per person**			1.6			0.9
<EUR 750	14.6%	117		9.9%	95	
EUR 750– <EUR 1500	57.3%	458		53.3%	511	
≥EUR 1500	28.0%	224		36.7%	352	
**Socioeconomic status in childhood**			1.6			1.8
Low	57.2%	457		55.1%	523	
Middle	37.4%	299		39.5%	375	
High	5.4%	43		5.5%	52	
**Number of chronic conditions**			0			0
0	40.8%	331		44.9%	434	
1	33.3%	270		30.0%	290	
2	15.5%	126		16.4%	159	
3+	10.5%	85		8.7%	84	

**Table 2:** Heath behaviour of parents and non-parents, stratified by sex (mean with 95% CI or proportion in %), N = 1779.

Variable	Mothers	N	Non-Mothers	N	Fathers	N	Non-Fathers	N
**Physical activity**								
Active in sports	43.2%	310	39.4%	37	31.9%	277	19.2%	19
Sport index	2.37 (2.32; 2.42)	715	2.33 (2.18; 2.47)	93	2.41 (2.36; 2.46)	864	2.09 (1.96; 2.21)	98
Leisure-time index	3.16 (3.11; 3.20)	717	3.11 (2.99; 3.23)	93	3.13 (3.08; 3.17)	867	3.10 (2.97; 3.23)	99
**Dietary pattern**								
Dietary pattern index	16.44 (16.08; 16.79)	717	16.49 (15.40; 17.58)	95	14.58 (14.34; 14.82)	866	14.44 (13.72; 15.15)	99
**Alcohol**								
0 g Alcohol per day	55.1%	395	54.7%	52	21.4%	185	21.2%	21
**Smoking**								
Current	15.2%	109	10.5%	10	22.5%	195	30.3%	30
Former	18.0%	129	11.6%	11	51.9%	450	46.5%	46
Never	66.8%	479	77.9%	74	25.6%	222	23.2%	23

**Table 3:** Coefficients (β) and 95% confidence intervals (95% CIs) from linear regression analyses indicating associations between parental status and health behaviours for women and men.

Outcome Variable	Unadjusted	Adjusted *
	β (95%CI)	β (95%CI)
**Women**		
Sport index	0.05 (−0.11; 0.20)	0.10 (−0.06; 0.25)
Leisure-time index	0.05 (−0.08; 0.18)	0.06 (−0.07; 0.20)
Dietary pattern index	−0.28 (−0.95; 0.40)	−0.05 (−0.76; 0.66)
**Men**		
Sport index	0.33 (0.18; 0.48)	0.29 (0.14; 0.44)
Leisure-time index	0.03 (−0.11; 0.16)	0.03 (−0.11; 0.16)
Dietary pattern index	−0.08 (−0.75; 0.58)	−0.04 (−0.71; 0.62)

* Adjusted for age, partner, years of education, occupational position, income, childhood socioeconomic status and number of chronic conditions.

**Table 4:** Odds ratios (ORs), 95% confidence intervals (95% CIs) from binomial and multinomial logistic regression analyses indicating associations between parental status and health behaviours for women and men.

Outcome Variable	Unadjusted	Adjusted *
	OR (95%CI)	OR (95%CI)
**Women**		
**Active in sports**	1.17 (0.76; 1.82)	1.32 (0.82; 2.14)
**Consumption of alcohol**	0.99 (0.64; 1.52)	0.84 (0.52; 1.36)
**Smoking status**		
Former vs. current smoker	1.08 (0.44; 2.63)	1.60 (0.61; 4.21)
Never vs. current smoker	0.59 (0.30; 1.19)	0.99 (0.46; 2.12)
**Men**		
**Active in sports**	1.98 (1.18; 3.33)	2.06 (1.17; 3.61)
**Consumption of alcohol**	0.99 (0.60; 1.65)	0.89 (0.52; 1.53)
**Smoking status**		
Former vs. current smoker	1.51 (0.92; 2.46)	1.35 (0.77; 2.38)
Never vs. current smoker	1.49 (0.84; 2.64)	1.45 (0.76; 2.78)

* Adjusted for age, partner, years of education, occupational position, income, childhood socioeconomic status and number of chronic conditions.

## Data Availability

The data presented in this study are available on request from the corresponding author.
